# Understanding and modifying Fabry disease: Rationale and design of a pivotal Phase 3 study and results from a patient-reported outcome validation study

**DOI:** 10.1016/j.ymgmr.2022.100862

**Published:** 2022-03-26

**Authors:** Christoph Wanner, Virginia Kimonis, Juan Politei, David G. Warnock, Nurcan Üçeyler, Aline Frey, Peter Cornelisse, Derralyn Hughes

**Affiliations:** a Department of Medicine, Division of Nephrology, University Hospital Würzburg, Oberduerrbacher Str. 6, 97080 Würzburg, Germany; b Division of Genetics and Genomic Medicine, Department of Pediatrics, University of California, Irvine, 333 The City Blvd. West, Suite 800, Orange, CA 92868, USA; c Foundation for the Study of Neurometabolic Diseases, FESEN, Uriarte 2383, C1425 CABA, Buenos Aires, Argentina; d University of Alabama at Birmingham, 1720 University Blvd, Birmingham, AL 35294, USA; e Department of Neurology, University Hospital of Würzburg, Oberduerrbacher Str. 6, 97080 Würzburg, Germany; f Idorsia Pharmaceuticals Ltd, Hegenheimermattweg 91, 4123 Allschwil, Switzerland; g University College London and Royal Free Hospital, Pond Street, London NW3 2QG, United Kingdom

**Keywords:** Lucerastat, Fabry disease, MODIFY, Substrate reduction therapy, α-GAL A, lysosomal enzyme α-galactosidase, AE, adverse event, b.i.d., twice daily, BPI-SF, Brief Pain Inventory-Short Form, BPI-SF3, Brief Pain Inventory-Short Form item 3, BSS, Bristol stool scale, CD, cognitive debriefing, CE, concept elicitation, CESD-R-20, Center for Epidemiologic Studies Depression Scale Revised, CKD-EPI, Chronic Kidney Disease Epidemiology Collaboration, CTCAE, Common Terminology Criteria for Adverse Events, ECG, electrocardiography, eGFR, estimated glomerular filtration rate, EOS, end of study, EOT, end-of-treatment, ERT, enzyme replacement therapy, FABPRO-GI, FABry Disease Patient-Reported Outcome-GastroIntestinal, FD, Fabry disease, FGID, functional gastrointestinal disorders, Gb3, globotriaosylceramide, GCS, glucosylceramide synthase, GI, gastrointestinal, GSRS, Gastrointestinal Symptom Rating Scale, HbA1c, hemoglobin A1c, IBS, irritable bowel syndrome, IRB, independent review board, LVEF, left ventricular ejection fraction, LVMI, left ventricular mass index, NeP, neuropathic pain, NPSI, neuropathic pain symptom inventory, NRS-11, 11-point numerical rating scale, NYHA, New York Heart Association, OLE, open-label extension, PGIC-DS, Patient Global Impression of Change in Disease Severity, PGIC-PS, Patient Global Impression of Change in neuropathic Pain Severity, PGIS-D, Patient Global Impression of Severity of Disease, PGIS-P, Patient Global Impression of Severity of neuropathic pain, PK, pharmacokinetics, PRO, patient-reported outcome, SD, standard deviation, SF-36v2, 36-Item Short Form Health Survey Version 2, SRT, substrate reduction therapy, UCI, University of California, Irvine, UT, usability testing

## Abstract

The use of available treatments for Fabry disease (FD) (including enzyme replacement therapy [ERT]) may be restricted by their limited symptom improvement and mode of administration. Lucerastat is currently being investigated in the MODIFY study as oral substrate reduction therapy for the treatment of FD. By reducing the net globotriaosylceramide (Gb3) load in tissues, lucerastat has disease-modifying potential to improve symptoms and delay disease progression.

MODIFY is a multicenter, double-blind, randomized, placebo-controlled, parallel-group Phase 3 study (ClinicalTrial.gov: NCT03425539); here we present the rationale and design of this study. Eligible adults with a genetically confirmed diagnosis of FD and FD-specific neuropathic pain entered screening. Patients were randomized (2:1) to receive either oral lucerastat twice daily or placebo for 6 months; treatment allocation was stratified according to sex and ERT treatment status. The main objectives of MODIFY are to assess the effects of lucerastat on neuropathic pain, gastrointestinal (GI) symptoms, FD biomarkers, and determine its safety and tolerability.

Neuropathic pain and GI symptoms are key features of FD that have a significant impact on quality of life. Despite various tools available to assess pain and GI symptoms, there are currently limited tools available to assess neuropathic and GI symptoms in FD, validated according to health authority guidelines. Based on FDA recommendations, we undertook a patient-reported outcome (PRO) validation study, using a novel eDiary-based PRO tool to assess the validity of evaluating neuropathic pain as a primary efficacy endpoint in MODIFY. Results from the PRO validation study are included.

To date, MODIFY is the largest Phase 3 clinical study conducted in patients with FD. Enrollment to MODIFY is now complete, with 118 patients randomized. Results will be presented in a separate publication. Long-term effects of lucerastat are being assessed in the ongoing open-label extension study (NCT03737214).

## Highlights

• There remains a need for treating patients with Fabry disease.
• MODIFY investigated lucerastat as substrate reduction therapy for Fabry disease.
• Limited tools are available to assess neuropathic and GI symptoms in Fabry disease.
• MODIFY assessed neuropathic pain and GI symptoms as primary and secondary efficacy endpoints.
• MODIFY used FDA-recommended validated tools for primary and secondary efficacy endpoints.

## 1 Introduction

Fabry disease (FD) is a rare lysosomal storage disorder caused by a deficiency in lysosomal enzyme α-galactosidase (α-GAL A) [1], which leads to progressive accumulation of globotriaosylceramide (Gb3) and subsequent manifestation of clinical symptoms [2]. FD phenotypes range in severity [3] and, if left untreated, may lead to progressive damage in vital organ systems [4,5].

Neuropathic pain is a key symptom of FD that significantly impacts quality of life in both male and female patients [6,7]. Despite the use of enzyme replacement therapy (ERT), 80% of patients with FD still exhibit clinical symptoms of neuropathic pain [8]. Various tools to assess pain severity have been developed and validated in other indications [[9], [10], [11], [12], [13], [14]], with only a limited number of questionnaires specific to FD emerging for potential use in clinical trials and real-world settings [[15], [16], [17], [18]]. Furthermore, according to the latest FDA recommendations, such questionnaires are not suitable to assess neuropathic pain as a primary efficacy endpoint in clinical studies [19]. Gastrointestinal (GI) involvement (e.g., diarrhea, abdominal pain, constipation) is the second most common feature of FD, affecting approximately 50–60% of patients with FD [4,20]. Due to the overlap of symptoms between FD and functional GI disorders (FGID) [21], and in line with FDA recommendations for developing drug treatments for treatment of FD, existing FDA-recommended patient-reported outcome (PRO) tools for irritable bowel syndrome (IBS) could be used to assess GI symptoms in FD [19,22]. Therefore, in addition to the unmet medical need in FD, selection of meaningful endpoints and their appropriate measurement by regulatory-acceptable tools are important considerations in the design of clinical studies for FD.

Lucerastat is a novel inhibitor of glucosylceramide synthase (GCS) in development as oral substrate reduction therapy (SRT) for the treatment of FD [23]. In Fabry patient fibroblasts, lucerastat resulted in a decrease of Gb3 and lysosomal staining irrespective of mutation type [24]. In an exploratory study, lucerastat 1000 mg twice daily reduced mean Gb3 levels by 55% (standard deviation: 10.4) at Week 12 in patients with FD receiving ERT [25].

Lucerastat oral monotherapy is currently being investigated in the pivotal Phase 3 MODIFY study. Here, we describe the intent and design of the MODIFY study. We also present results of a validation study that evaluated the content validity of an eDiary-based PRO tool to assess neuropathic pain and GI symptoms as primary and secondary efficacy endpoints, respectively, in MODIFY.

## 2 Materials and methods

### 2.1 MODIFY study design

**Fig. 1:**
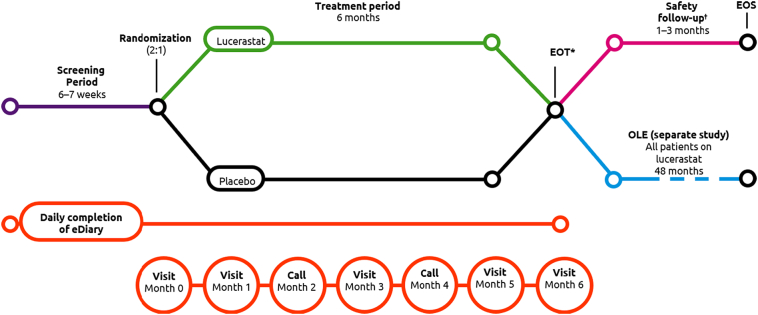
MODIFY study design. *For patients who enter the OLE study, the EOS corresponds to the EOT visit. ^†^The safety follow-up is applicable to all patients except those who enter the OLE study. EOS, end of study; EOT, end-of-treatment; OLE, open-label extension.

MODIFY is a **M**ulticenter, d**O**uble-blind, ran**D**omized, placebo-controlled, parallel-group Phase 3 study to determine the clinical eff**I**cacy and safety of lucerastat oral monotherapy in adult patients with **F**abr**Y** disease. The study is comprised of a screening period lasting 6**–**7 weeks and a double-blind treatment period lasting 6 months. Patients who complete the 6-month treatment period have the option to enter into an open-label extension study (conducted under a separate protocol, ClinicalTrials.gov: NCT03737214); patients who do not enter the extension study continue to the safety follow-up period (Fig. 1), which lasts 1 month for female patients and 3 months for male patients. MODIFY is being conducted in full compliance with the principles of the Declaration of Helsinki, International Conference on Harmonization Good Clinical Practice Guidelines, and local laws and regulations of the countries in which the study is conducted. MODIFY is registered on Clinicaltrials.gov (NCT03425539).

#### 2.1.1 Specific objectives of MODIFY

The primary objective is to determine the effect of lucerastat on neuropathic pain in patients with FD. Secondary objectives are to: 1) determine the effects of lucerastat on GI symptoms in patients with GI symptoms at baseline; 2) confirm the effect of lucerastat on biomarkers of FD; and 3) determine the safety and tolerability. Other objectives include the evaluation of lucerastat on renal function, cardiac parameters, depression, and quality of life and the documentation of the pharmacokinetics (PK) of lucerastat.

#### 2.1.2 MODIFY study population and randomization

MODIFY specifically enrolled adult patients with a diagnosis of FD (confirmed by the presence of a *GLA* mutation) and Fabry-associated neuropathic pain in the 3 months prior to screening. Full inclusion and exclusion criteria are listed in Table 1. A total of approximately 99 eligible patients were planned to be randomized (2:1) to lucerastat or placebo. Treatment allocation was stratified by sex (male/female) and by ERT treatment status at screening (treated, “pseudo-naïve”/“treatment-naïve”). The targeted population of patients with FD is homogenous, based on the criteria for Fabry-associated neuropathic pain. Published guidelines on neuropathic pain recommend studying the efficacy of a treatment in a homogenous population with respect to diagnosis and stratifying according to baseline disease characteristics [26].

**Table 1:** Inclusion and exclusion criteria for MODIFY.

Inclusion criteria	
	*Screening visit*
1	Signed and dated informed consent form, provided prior to any study-mandated procedure
2	Male or female patients aged 18 years old and above
3	FD diagnosis confirmed with local genetic test results (i.e., presence of at least 1 mutation in *GLA*, the gene coding for α-GAL A)
4	Fabry-associated neuropathic pain, as defined by the patient, in the last 3 months prior to screening
5	ERT treatment status: a) Never treated with ERT; or b) Not received ERT for at least 6 months prior to screening; or c) ERT at the time of the screening visit, and meeting all of the following criteria at screening: i) ERT administration for the last 12 months; ii) Stable ERT dose regimen during the last 3 months; iii) Patient agrees to stop ERT administration at the screening visit for approximately 8 months (6–7 weeks screening + 6 months of double-blind treatment)
6	Women of childbearing potential are eligible if: • They have a negative pregnancy test at screening and a negative urine pregnancy test at randomization • They agree to undertake monthly urine pregnancy tests during the study (and follow a highly effective contraception scheme from screening) up to at least 30 days after study drug discontinuation • They agree to follow a highly effective contraception scheme from screening up to at least 30 days after study treatment discontinuation
7	A fertile male (physiologically capable of conceiving a child according to investigator judgment) who is sexually active with a woman of childbearing potential is eligible only if the following applies: • Agreement to use a condom during the treatment period (starting at randomization) and for up to 3 months after study treatment discontinuation; and • Agreement not to father a child during this period
	*Randomization visit*
8	Adequate patient compliance with completion of an eDiary during the screening period
9	Patients with moderate or severe^a^ neuropathic pain as determined from daily entries of the modified BPI-SF3 score of “neuropathic pain at its worst in the last 24 h” in the eDiary during the screening period


Exclusion criteria	
	*Screening visit*
1	Pregnant/planning to become pregnant up to 30 days after study treatment discontinuation, or lactating patient
2	Severe renal insufficiency defined as an eGFR per the CKD-EPI creatinine equation <30 mL/min/1.73 m^2^ at screening (as reported by the central laboratory)
3	On regular dialysis for the treatment of chronic kidney disease
4	Patient has undergone, is on a waiting list for, or is scheduled to undergo kidney or other organ transplantation
5	Known and documented transient ischemic attack, stroke, unstable angina, or myocardial infarction within 6 months prior to screening
6	Clinically significant unstable cardiac disease in the opinion of the investigator (e.g., uncontrolled symptomatic arrhythmia, NYHA class III or IV congestive heart failure)
7	Any other patient at high risk of developing clinical signs of organ involvement within the time period of the study, as per investigator judgment
8	Any known factor or disease that might interfere with treatment compliance, study conduct or interpretation of the results such as: • Other disease or condition associated with a pain component that could confound assessment of neuropathic pain (e.g., diabetic neuropathy, chemotherapy- or radiation-induced peripheral neuropathy, chronic inflammatory demyelinating polyneuropathy) • Other disease of the GI tract that could interfere with the assessment of GI symptoms in FD (e.g., inflammatory bowel disease, galactose intolerance, total lactase deficiency or glucose-galactose malabsorption) • Documented poorly controlled diabetes mellitus (i.e., HbA1c >8.0% at screening as reported by the central laboratory) • Significant neurological disorder • Significant psychiatric disease; suicidal ideation at screening or history of suicide attempt or behavior within 6 months prior to screening as per investigator judgment • History of drug dependence (including opioids) or alcohol dependence • Inability to complete an eDiary on a daily basis
9	Known concomitant life-threatening disease with a life expectancy <18 months
	*Treatments*
10	Patient planned for imminent initiation of treatment with ERT
11	Known hypersensitivity to lucerastat or drug of the same chemical class of iminosugars (e.g., miglitol, miglustat, migalastat), or any of their excipients (including lactose)
12	Initiation or treatment at an unstable dose within 4 weeks prior to screening with any of the following medications: • ACE inhibitor and/or ARB • Anti-epileptic • TCA and/or other antidepressants belonging to SNRI and selective serotonin re-uptake inhibitor classes
13	Planned or current treatment with another investigational treatment within 3 months prior to screening
14	Treatment with any inhibitor of GCS (e.g., miglustat, lucerastat, eliglustat, ibiglustat/venglustat) or an α-Gal A chaperone (e.g., migalastat) within 6 months prior to screening
	Randomization visit^b^
10	Treatment with ERT (agalsidase alfa, agalsidase beta) during the screening period

α-GAL A, alpha galactosidase A; ACE, angiotensin-converting enzyme; ARB, angiotensin receptor blocker; BPI-SF3, Brief Pain Inventory-Short Form item 3; CKD-EPI, Chronic Kidney Disease Epidemiology Collaboration; eGFR, estimated glomerular filtration rate; ERT, enzyme replacement therapy; FD, Fabry disease; GCS, glucosylceramide synthase; GI, gastrointestinal; HbA1c, hemoglobin A1c; NYHA, New York Heart Association; SNRI, serotonin-norepinephrine re-uptake inhibitor; TCA, tricyclic antidepressant.

a Moderate or severe neuropathic pain during the screening period is defined based on average modified BPI-SF3 score of “neuropathic pain at its worst in the last 24 h” from daily entries in an eDiary during the 4 weeks preceding randomization.

b Investigators must verify that patients do not fulfill the exclusion criteria checked at screening (as applicable).

#### 2.1.3 MODIFY study medication

Patients receive either lucerastat or matching placebo orally (capsules), twice daily. The starting dose of lucerastat is based on a patient's estimated glomerular filtration rate (eGFR) value at screening. During the study, the dose of lucerastat is adjusted if a patient's eGFR crosses boundary thresholds, defined in Table 2. Patients initiate treatment on the evening of the randomization visit and, thereafter, take each treatment dose in the morning and evening irrespective of food intake (preferably at the same time). On the morning of study visit days, patients should not take study treatment, which is only administered after completion of pre-dose assessments.

**Table 2:** eGFR-based study treatment dosing scheme.

eGFR (mL/min/1.73 m^2^)	Dose regimen, mg b.i.d. (oral)	Number of capsules per dosing
≥60	1000	4
≥45 and < 60	750	3
≥30 and < 45	500	2
≥15^a^ and < 30	250	1

b.i.d., twice daily; CTCAE, Common Terminology Criteria for Adverse Events; eGFR, estimated glomerular filtration rate.

a Study treatment must be stopped if eGFR <15 mL/min/1.73 m^2^ or in the event that the acute kidney injury CTCAE grade 2 or above is met.

Patients will permanently discontinue study treatment at any time during the study: if eGFR <15 mL/min/1.73 m^2^ or in the event of acute kidney Common Terminology Criteria for Adverse Events (CTCAE) grade 2 or above; in the event of heart failure leading to in-patient hospitalization or prolongation of hospitalization; in the event of stroke CTCAE grade 3 or above; or if a patient becomes pregnant while on study treatment.

#### 2.1.4 MODIFY key study endpoints

The results from a novel PRO validation study (see 2.2) were used to inform the suitability of the primary efficacy endpoint, which is the response to lucerastat on neuropathic pain, defined as a change from baseline to Month 6 in the “modified” Brief Pain Inventory-Short Form item 3 (BPI-SF3) score of “neuropathic pain at its worst in the last 24 h”. Secondary efficacy endpoints are the change from baseline to Month 6 in: 1) plasma Gb3; 2) average daily 11-point numerical rating scale (NRS-11) score of “abdominal pain at its worst in the last 24 h” in patients with GI symptoms at baseline; and 3) the number of days with at least one stool of Bristol stool scale (BSS) consistency Type 6 or 7 in patients with GI symptoms at baseline. Other efficacy endpoints, including renal function and echocardiography endpoints, are summarized in Table 3. Long-term outcome of renal function and echocardiography parameters will be further assessed in the open-label extension study.

**Table 3:** Summary of other efficacy endpoints.^a^

Renal function endpoints	
	Patient eGFR slope from baseline to Month 6
	Changes from baseline to Month 6 in urine albumin-to-creatinine ratio
Echocardiography-based endpoints	
	Changes from baseline to Month 6 in LVMI, posterior wall thickness, left ventricular mean wall thickness, LVEF, left ventricular end diastolic and end systolic volumes, left atrial volume
Pain medication endpoints based on daily eDiary entries	
	Patient mean weekly dose of opioid analgesics from baseline to Month 6
	Use of significant rescue pain therapy from baseline to Month 6
	Total number of days on significant rescue pain therapy from baseline to Month 6
Clinical symptoms endpoints based on data collected at site visits	
	Change from baseline to Month 6 in the subject's rating of item 5 score of the BPI-SF (“pain on the average in the last 24 h”)
	Change from baseline to Month 6 in the total score of the subject's rating of item 9 of the BPI-SF (7 pain interference questions: “general activity”, “mood”, “walking ability”, “normal work”, “relation with other people”, “sleep”, “enjoyment of life”)
	Change from baseline to Month 6 in patient's rating of severity of NeP, as measured by PGIS-P
	Patient's rating of change in overall severity of NeP since study treatment start, as measured by PGIC-PS at Month 6
	Change from baseline to Month 6 in patient's rating of disease severity, as measured by PGIS-D
	Patient's rating of change in disease severity since study treatment start, as measured by PGIC-DS at Month 6
	Change from baseline to Month 6 in total score of patient's rating of CESD-R-20
Treatment failure	
	Time to treatment failure from baseline to Month 6^b^

BPI-SF, Brief Pain Inventory-Short Form; CESD-R-20, Center for Epidemiologic Studies Depression Scale Revised; eGFR, estimated glomerular filtration rate; LVEF, left ventricular ejection fraction; LVMI, left ventricular mass index; NeP, neuropathic pain; PGIC-DS, Patient Global Impression of Change in Disease Severity; PGIC-PS, Patient Global Impression of Change in neuropathic Pain Severity; PGIS-D, Patient Global Impression of Severity of Disease; PGIS-P, Patient Global Impression of Severity of neuropathic pain.

a Endpoints are assessed from baseline to Month 6, unless otherwise stated.

b Defined as initiation or re-initiation of ERT, or permanent study discontinuation for any reason.

##### 2.1.4.1 Amendment to the methodology to analyze the primary efficacy endpoint

Based on early interaction in 2016 and 2017 with FDA, and prior to commencement of MODIFY, a dichotomous responder-based analysis for the primary efficacy endpoint was described in the protocol (response to study treatment on neuropathic pain, defined as a reduction from baseline to Month 6 of at least 30% in the “modified” BPI-SF3 score of “neuropathic pain at its worst in the last 24 h”). In early 2020, further interaction with FDA led to a methodological change of the analysis of the primary efficacy endpoint to a continuous analysis (change from baseline to Month 6 in the “modified” BPI-SF3 score of “neuropathic pain at its worst in the last 24 h”).

Analyzing the primary efficacy endpoint as a continuous variable avoids a loss in information caused by the dichotomization of the endpoint variable and, therefore, requires fewer patients to maintain the statistical power to detect a difference between lucerastat and placebo.

### 2.2 Validating primary efficacy and secondary efficacy endpoints for MODIFY: a PRO validation study

In clinical studies, the NRS-11 “pain at its worst in the last 24 h” has been used to assess pain severity; the use of an NRS is supported by IMMPACT recommendations for assessing pain [27]. Despite the widespread use of the BPI-SF in FD, item 3 of the BPI-SF (BPI-SF3, “pain at its worst in the last 24 h”) is not specific to neuropathic pain in FD and has not been validated in this population. Additionally, since there is an overlap of GI symptoms experienced by patients with FD and patients with IBS, PRO tools (NRS-11 and BSS) recommended by the FDA IBS guidance could be used to assess GI symptoms in FD [22]. Therefore, a PRO validation study based upon a “modified” BPI-SF3 specific to FD (that included a definition of neuropathic pain) was developed, for use in assessing the primary efficacy endpoint in the MODIFY study.

We conducted a PRO validation study in patients with FD. The primary objectives were to spontaneously elicit definitions and descriptions of neuropathic pain experienced in FD and to cognitively debrief and test the patient acceptability of the PRO instruments on an electronic device.

#### 2.2.1 PRO validation study design

The PRO validation study comprised two parts: 1) concept elicitation (CE) telephone interviews to elicit definitions and descriptions of neuropathic pain associated with FD (Appendix A 1); and 2) face-to-face cognitive debriefing (CD) and usability testing (UT) interviews (Appendix A 2) to assess the content validity of the primary and secondary efficacy endpoint measures, test the usability of the eDiary, and to gain input about meaningful change from the patient perspective. Interviews were conducted by a specialized vendor, and the study was approved by the University of California, Irvine (UCI) independent review board (IRB) (UCI IRB HS#2017-3815).

#### 2.2.2 PRO validation study: patient eligibility

Adult (male and female) patients were required to have a diagnosis of FD (confirmed with genetic test results in the subject's medical history), Fabry-associated neuropathic pain for at least 3 months prior to study entry, and be fluent in US English (able to speak, read, write, and comprehend). Patients were not eligible if they had a disease with a pain component or disease of the gastrointestinal tract that could interfere with the assessment of neuropathic pain or GI symptoms, respectively.

#### 2.2.3 PRO validation study: procedures

Concept elicitation (CE) telephone interviews (lasting approximately 1 h) were audio-recorded and conducted using a semi-structured CE interview guide comprised of open-ended questions that encouraged spontaneous reporting of concepts from patients. Interview guides included topics, questions, and probes to understand how patients with FD experience neuropathic pain. Audio recordings were transcribed verbatim and anonymized, and each transcript was considered a unit of analysis. A coding scheme was applied and operationalized using ATLAS.ti version 7 and was initially developed based on the interview guide and study objectives. For CE interviews, the coding scheme catalogued concepts spontaneously reported by patients (without prompting from the interviewer), and the coding process was guided by grounded theory and constant comparative method. The coding scheme was updated as necessary to reflect actual terms patients used to describe concepts. Codes were applied to specific text within each transcript and then queries for frequency across transcripts.

Saturation was considered to be achieved at the point when additional interviews were unlikely to yield new information (i.e., new concepts of importance and relevance to patients) [28].

During CD interviews (audio-recorded and lasting approximately 90 min) patients were asked to complete an eDiary using a think-aloud method (patients were encouraged to verbalize their thoughts while completing the eDiary). Under this methodology, the interviewer simply reminds respondents to verbalize their thought process (e.g., “what were you considering when you selected that answer?”) [29]. The UT portion was also used to determine if patients had any difficulty using the eDiary device, and patients were asked to respond to structured rating questions related to device usability. Like the CE interviews, audio-recordings of the interviews were transcribed and coded using ATLAS.ti. version 7.0.

## 3 Results

The results of the PRO validation study are presented here. Fifteen patients with FD were screened from one site (UC Irvine), of which 13 were successfully recruited into the PRO validation study and participated in the CE interviews (two patients did not report experiencing neuropathic pain). Ten of the thirteen patients participated in the CD/UT interviews. Demographics were generally balanced between the two parts of the study (Table 4).

**Table 4:** Patient-reported demographics (PRO validation study).

	CE interviews (n = 13)	CD/UT interviews (n = 10)
Mean age, years (SD)	44.1 (11.7)	39.5 (8.7)
Sex, number (%)		
Male	7 (54)	6 (60)
Female	6 (46)	4 (40)
Race, number (%)		
White	7 (54)	6 (60)
Other^a^ (Mexican American)	4 (31)	3 (30)
Other^a^ (Spaniard)	2 (15)	1 (10)
Ethnicity, number (%)		
Hispanic/Latino	9 (69)	5 (50)
Not Hispanic/Latino	4 (31)	5 (50)

CD, cognitive debriefing; CE, concept elicitation; PRO, patient-reported outcome; SD, standard deviation; UT, usability testing.

a The “Other” response option gave patients the opportunity to fill in a specific response, and their write-in response is reported verbatim here.

**Fig. 2:**
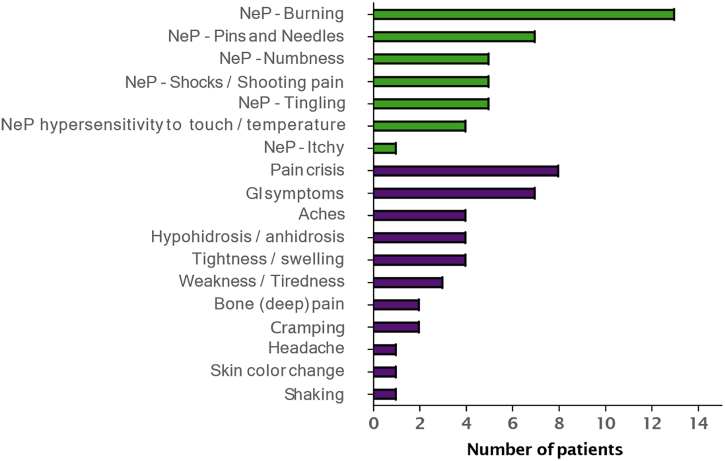
Overall symptoms described during CE interviews (PRO validation study). CE, concept elicitation; GI, gastrointestinal; NeP, neuropathic pain; PRO, patient-reported outcome.

**Fig. 3:**
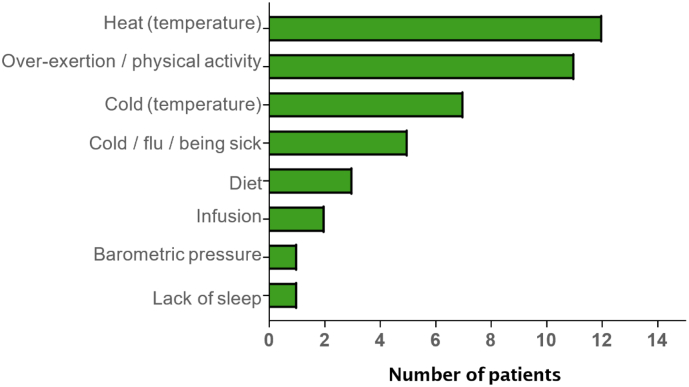
Neuropathic pain symptom triggers described during CE interviews (PRO validation study). CE, concept elicitation; PRO, patient-reported outcome.

Patients with FD shared their experience of FD when asked non‑leading questions, and the concepts elicited are summarized in Fig. 2. The most common symptoms described as part of the neuropathic pain experience were “burning” (“it feels like my hands are on fire… like I'm walking on hot coals”), “pins and needles” (“it feels like there's a thousand needles poking at my hands and feet, almost to where if you were to get out of bed… and I was to walk, it would feel like I'm walking on hot coals with needles jabbing into my feet”), “numbness” (“…where there's so much pain radiating through that it becomes numb so you can't walk on them because – or use your hands for whatsoever, because it's at that point where you know you don't have as well as control as you can, or you can't function as well as you normally would”), “shocks/shooting pain” (“if I moved even just a little bit, I would feel shocks just radiating throughout my whole body”), and “tingling” (“I can feel it begin to tingle, and then it burns”). Six of thirteen patients had heard of the term “neuropathic pain” and were able to define it or use it from the beginning. Two patients had heard of the term, but were not sure what it meant, while one patient had never heard of the term (four patients were not asked about the term). Patients were able to distinguish between a neuropathic pain experience versus other types of pain experiences. The most common triggers of neuropathic pain elicited by patients were “heat” (temperature), “overexertion/physical activity”, and “cold” (temperature) (Fig. 3). Pain crisis was a multidimensional concept that was experienced by eight patients, described in terms of its location (e.g., radiating beyond hands and feet), various sensations (e.g., aching [“aching, especially in the bones”], and shooting pains [“shooting pains throughout my body”]), and severity (e.g., extreme [“all those things that I told you to happen to me with the pain… all in one, but extreme”]). An analysis of concept occurrence demonstrated that saturation was reached for the total number of unique concepts and for neuropathic pain-related concepts. No new concepts emerged after the 9th interview (of 13 interviews) (Fig. 1S). All patients interpreted questions on neuropathic pain, abdominal pain, and bowel movement frequency as intended. Debriefing characteristics included adding relevant triggers (“being sick”, n = 7; “stress”, n = 5; “weather change”, n = 5; “diet”, n = 3; “tiredness”, n = 2) and changing “permanent pain” to “constant pain” (n = 4). On the questions of abdominal pain, four patients suggested changing “pain” to “pain/discomfort”.

**Fig. 4:**
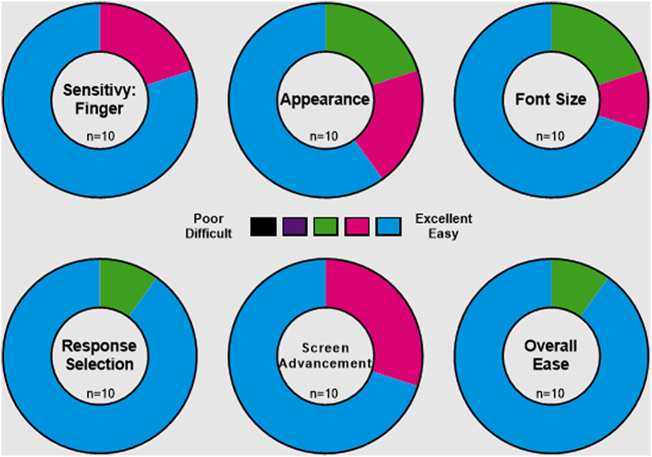
eDiary usability testing results.

The eDiary device demonstrated good usability among patients with FD. Ninety percent of patients rated the overall ease of use and ability to select an answer as a five (on a scale of 1 to 5) (Fig. 4). Overall, patients did not have difficulty interpreting the content of the FD eDiary.

### 3.1 PRO validation study: outcomes

Based on these findings, the wording of the neuropathic pain severity question for MODIFY was revised by adding additional descriptors and triggers (bolded) to the definition of neuropathic pain (“modified” BPI-SF3):

• “Please rate your neuropathic pain (e.g., pain that feels like burning, shocks or shooting, stabbing, tingling, and/or pins and needles) in your hands and feet by selecting the number that best describes your neuropathic pain at its WORST in the last 24 h”
  ▪ “The following question is about the neuropathic pain you may have because of your Fabry disease. This type of pain usually feels like burning, shocks, or shooting pain, tingling, pins and needles, stabbing, and/or numbness in the hands and feet. You may have **constant pain** of variable intensity. You may also get attacks of intense, excruciating pain that starts in the hands and feet and spreads to other parts of the body. Your pain may occur randomly. It may also be triggered by heat or cold, **weather change, being sick or having a fever, diet, stress, tiredness**, and/or physical activity”.

For the abdominal pain severity question, although four patients suggested rewording the concept to “pain/discomfort”, no changes were made since more recent IBS studies focus on pain and treat pain as conceptually distinct from abdominal discomfort. Furthermore, this is aligned with the FDA guidance on IBS studies [22].

### 3.2 MODIFY key measurements/assessments

The findings from the PRO validation study helped inform the suitability of assessing neuropathic pain as a primary endpoint within MODIFY; furthermore, the experience gained through employment of the eDiary helped shape how the eDiary could be used within MODIFY. During MODIFY, each patient was provided with a portable eDiary, trained to use the device by study personal during screening, and completed the eDiary according to the protocol schedule. All PRO assessments collected either on a daily basis (evening diary) or during a site visit were recorded in the eDiary. Patients rated their neuropathic pain (daily) from screening until Month 6. Neuropathic pain intensity was rated on an NRS-11 to respond to the modified BPI-SF3 in the eDiary. Patients rated (daily) their abdominal pain and the number and consistency of bowel movements from screening to Month 6. The patients' impression of disease severity was assessed by two Patient Global Impression of Severity (PGIS) questionnaires (PGIS-D, disease severity; PGIS-P, neuropathic pain severity) at each site visit. Two Patient Global Impression of Change (PGIC) questionnaires assessed the patient's impression of change since study treatment start in disease severity (PGIC-DS) and neuropathic pain (PGIC-PS) at each site visit. In addition, the full BPI-SF was completed by the patient at each site visit, while a quality-of-life questionnaire (36-Item Short Form Health Survey Version 2 [SF-36v2™]) and a depression scale (Center for Epidemiologic Studies Depression Scale Revised [CESD-R-20]) were completed by the patient both at randomization and Month 6. The eDiary was used to collect pain medication use.

Plasma and urinary levels of Gb3 and lysoGb3 were quantified at each site visit by a central laboratory. To minimize the possibility for systematic unblinding, results of FD biomarker assessments were not communicated to the study site or sponsor until the study database was locked. Renal function and echocardiography-based parameters were assessed from baseline to Month 6. Safety assessments included adverse events (AEs), serious AEs, physical examination, vital signs (heart rate, systolic and diastolic blood pressure), weight, and electrocardiography (ECG) assessment (12‑lead ECG) and laboratory evaluation of blood and urine.

### 3.3 MODIFY sample size, hypothesis, and statistical analysis

The planned sample size for MODIFY was originally 108 patients. However, with the change to the methodology used to analyze the primary efficacy endpoint (as determined via the PRO validation study), a sample size of approximately 99 patients provides 87.2% power to detect a treatment difference of 2 points, with an assumed standard deviation of 3 points (on the 11-point scale) primarily based on a test for the mean difference between lucerastat and placebo in the change from baseline to Month 6 in the “modified” BPI-SF3 score. Calculations were conducted using East 6.5 based on a two-sided *t*-test for independent samples.

The primary statistical approach will be performed according to the intent-to-treat approach. The Type I error rate will be controlled at a two-sided alpha of 5% for the testing of the four null hypotheses associated with the primary and secondary efficacy endpoint comparisons employing a fixed-sequence statistical testing strategy.

## 4 Discussion

To date, MODIFY is the largest pivotal Phase 3 study conducted in FD. The primary objective of MODIFY is to determine the effect of lucerastat on neuropathic pain in patients with FD. Safety, as well as the effect of lucerastat on other key symptoms of FD, will also be assessed.

Current management of FD consists of conventional symptomatic and supportive managements, ERT, and chaperone therapy. Despite the use of ERT, a large proportion of patients still exhibit clinical symptoms, and improvements in neuropathic pain are limited [[30], [31], [32]]. Further, the long-term effects of ERT on risk of morbidity and mortality related to FD remain to be established [33]. Oral administration of migalastat is restricted to patients with amenable mutations, estimated to be 30–50% of patients with FD [34]. Overall, there is still an unmet medical need in the treatment of patients with FD. Lucerastat, a small molecule GCS inhibitor, provides a new mechanism of action (SRT) to be evaluated for the treatment of FD. The goal of SRT is to reduce the rate of synthesis of Gb3 to a level compatible with residual clearance. In mouse models, oral lucerastat monotherapy penetrated the liver, kidneys, heart, brain, and dorsal root ganglia; Gb3 was reduced in the kidneys and liver by more than 30% [35,36]. In cultured fibroblasts from patients with FD, including those with mutations associated with classical phenotype and those expected to have residual α-GAL A activity, lucerastat dose-dependently reduced levels of Gb3 [24]. Therefore, oral lucerastat has disease-modifying potential to alleviate FD-associated symptoms, irrespective of FD mutation, and delay disease progression.

MODIFY is the first clinical study in patients with FD to measure neuropathic pain as a primary endpoint, which uses an FDA-agreed PRO instrument to collect symptom information on a daily basis. Previously, neuropathic pain (or simply pain) has been assessed as a secondary or tertiary endpoint in most FD clinical studies, and only once as a primary endpoint in a single study [30]. There are various tools to assess general pain severity, which comprise non-specific questionnaires that have been developed and validated in other indications and include the BPI-SF [9,10], SF-36 [[11], [12], [13]], and the McGill pain questionnaire [14]. There are also questionnaires specific to FD (neuropathic pain symptom inventory [NPSI] [15] and the Fabry Pain Questionnaire [FPQ] [17,18]) but they are not suitable for use on a daily basis or to assess neuropathic pain as a primary endpoint in clinical studies. In line with guidance from The Committee for Medicinal Products for Human Use (CHMP) and the FDA, the “modified” BPI-SF3 is a unidimensional neuropathic pain scale that can be used in clinical studies assessing neuropathic pain as a primary efficacy endpoint in adult patients with FD. By providing a specific and adequate definition of neuropathic pain, it is expected that patients with FD will understand the concept in an accurate and intended manner. Furthermore, the “modified” BPI-SF3 is a tool that can be used on a daily basis using an electronic device.

GI symptoms in patients with FD can be episodic, alternating between diarrhea and periods of normal (and sometime reduced) bowel activity, and often mimic symptoms of FGID, such as IBS. Although the Gastrointestinal Symptom Rating Scale (GSRS), a PRO developed in other indications, has been previously used in FD studies, the recall period (7 days) is too long to capture symptoms that fluctuate on a daily basis [37]. The FABry Disease Patient-Reported Outcome-GastroIntestinal (FABPRO-GI) instrument was recently developed to specifically assess abdominal pain, diarrhea, and other GI symptoms in patients with FD but has, so far, not been evaluated in clinical trials [38]. Based on FDA advice to assess diarrhea and abdominal pain using PRO tools recommended by the FDA IBS guidelines [22], an NRS-11 to assess abdominal pain and the BSS to assess number and consistency of bowel movements were tested for use in patients with FD. Both tools were interpreted as intended by patients with FD, validating the use of these FDA-recommended tools to assess specific GI symptom endpoints in patients with FD.

The MODIFY study includes suitable and validated tools to assess primary and secondary endpoints for use in patients with FD, in line with health authority guidelines on endpoints in FD clinical studies. Study enrollment was completed in December 2020 with 118 patients randomized. Patients who complete MODIFY will have the option to enroll into an open-label extension study (NCT03737214), which will assess the long-term safety and tolerability of lucerastat, as well as the long-term effects on renal and cardiac function.

## Funding source

The MODIFY study was supported by Idorsia Pharmaceuticals Ltd.

## Appendix A Supplementary data

Appendix A Supplementary data Image 1
