# Supplementary material for: Understanding and modifying Fabry disease: Rationale and design of a pivotal Phase 3 study and results from a patient-reported outcome validation study
Source: Mol Genet Metab Rep. 2022 Mar 26;31:100862. doi: 10.1016/j.ymgmr.2022.100862 (PMC9248229; doi:10.1016/j.ymgmr.2022.100862)
Supplement: Supplementary file 1 — Appendix A Supplementary data [file mmc1.docx]

**Supplementary Materials**

**S1 Questions relating to the signs and symptoms of FD posed to patients during the concept elicitation (CE) interviews**

- How do you refer to your condition? Let’s say you are explaining it to a friend or family. What do you call it? Do you call it Fabry disease (FD)?
- What is it like to have FD? What are your symptoms?
- What is a typical day for you in terms of your FD?
  - What would you consider a “bad day” in terms of your FD?
  - What would you consider a “good day” in terms of your FD?
- In addition to the symptoms you already told me about, do you experience any other symptoms of FD?
- Can you tell me more about how the pain feels? (Listen closely for different descriptors like burning, tingling, stabbing, shooting, etc.)
  - How would you describe the pain to someone who has never experienced it?
    - Have you heard of the term “neuropathic pain”? [If yes]
    - How would you define it? [If no]
- When you hear that term, what do you think it means?
  - Where do you experience the pain ?
- Do you experience any other types of pain? Is the pain associated with FD the same or different than other types of pain (e.g., let’s say when you fall and cut yourself)? How is it different?
- Does the pain occur at a certain time of day? Under certain circumstances? Are there specific things that trigger the pain? Is there a pattern?
- How often does pain occur (e.g., number of days per week/month…)?
  - Within a day? Within a week? Within a month?
  - How long does the pain last? After a pain episode, how quickly does the pain return?
- How long does the pain last?
- How bad does the pain get at its worst?
  - On a scale of 0 to 10, where 0 is no pain and 10 pain as bad as you can imagine, how bad does it get on average? At its worst?
- Do you take any medications for your neuropathic pain? If so, what are they? Do they work and if so, how much do you feel better after taking it?
- Thinking about improvement in your pain, what would need to improve for it to be meaningful to you?
  - How often you have pain?
  - How long the pain lasts (i.e., how quickly the medication works and relieves pain)?
  - How bad the pain is?
- Let’s think back to your neuropathic pain at its worst. You rated that, at its worst, it can get up to [number from 0-10]. Let’s say there is a new treatment that can bring down that number. At which number from 0 to 10 would you say the drug is working and that you’re feeling better? (e.g., In the 0-3 range? In the middle at 5? etc.)
- Now, I am going to ask you on a 0-10 scale what would a small, moderate and a large improvement in neuropathic pain would mean to you. On a 0-10 scale:
  - How many points would you have to change to experience a small improvement that would be meaningful to you?
  - How many points would you have to change to experience a moderate improvement that would be meaningful to you?
  - How many points would you have to change to experience a large improvement that would be meaningful to you?

**S2 Questions posed to patients during the face-to-face cognitive debriefing (CD) and usability testing interviews**

**S2.1 Questions posed to patients during the face-to-face CD**

Screen 1

- What do you think these instructions are telling you here? Can you put it in your own words?
  - When it describes pain usually feeling like “burning, stabbing, tingling, and/or shooting pain”, which ones describe you? Can you explain?
  - What does “permanent pain” mean to you?
  - What does “variable intensity” mean to you?
  - What does it mean when it says “you may also get attacks of intense, excruciating, burning pain that starts in the hands and feet and spread out to other parts of the body”?
    - What does “intense, excruciating, burning” pain mean to you?
    - When it specifies the area of neuropathic pain as starting “in the hands and feet”, is this where your neuropathic pain usually starts? Where does the pain spread to?
  - What does it mean when it says “your pain may be triggered by e.g., heat, fever and/or physical activity”?
    - How do you define a “trigger”?
    - What are some other triggers of neuropathic pain for you? What other examples could we include that applies to you?
  - What does it mean when it says “it may also occur spontaneously”?
    - What is “it” referring to here? How would you define “spontaneously” here?
- Was it clear or unclear to you what to do from reading Screen 1? If unclear, why was it unclear?
- Would you reword or change anything about the instructions? Please explain.

Screen 2

- What does this question mean to you? Can you put the question into your own words?
  - I know we talked about neuropathic pain because of your FD during the telephone interviews, but can you please tell me again how you would define “neuropathic pain”?
  - What does “pain at its WORST” mean to you?
  - What does “in the last 24 hours” mean to you?
- Is the meaning of the question clear or unclear to you? If unclear, why is it unclear?
- I see you selected [patient’s response]. What made you choose that answer?
  - What does [response selected] mean to you? What were you considering when you answered the question? How were you feeling in the past 24 hours that made you select that answer?
  - How often would you select this number to describe your neuropathic pain? Does it fluctuate from day to day? Week to week? In different seasons? What makes it fluctuate?
  - You currently selected [patient’s response]. Let’s say there’s a drug that will decrease your neuropathic pain. What would be a meaningful improvement for you, i.e., if you moved down one number? Two numbers? Three?
  - Can you remember back to a time when you would have selected something other than zero? Thinking of this time, what number would you have selected and why?

Screen 3

- What does this question mean to you? Can you put the question into your own words?
  - What does “abdominal pain at its worst” mean to you?
- Is the meaning of the question clear or unclear to you? If unclear, why is it unclear?
- I see you selected [patient’s response]. What made you choose that answer?
  - What does [response selected] mean to you?
  - What were you considering when you answered the question?
- What does [insert response option] mean for you?
  - How often do you experience abdominal pain? Can you remember back to a time when you would’ve selected something higher than [patient’s response]? Something lower than [patient’s response]? How often does it fluctuate? What makes it change do you think?
  - You currently selected [patient’s response]. Let’s say there’s a drug that will decrease your abdominal pain. What would be a meaningful improvement for you, i.e., if you moved down one number? Two numbers? Three?
  - Has there ever been a time where you experience abdominal pain and would have selected another response?
    - [If yes, ask]: How often do you experience [concept]?

Screen 4

- What does this question mean to you? Can you put the question into your own words?
  - What does “bowel movement” mean to you?
- Is the meaning of the question clear or unclear to you? If unclear, why is it unclear?
- I see you selected [patient’s response]. What made you choose that answer?
  - Is coming up with this number in the past 24 hours difficult or easy? If difficult, what is difficult about it?
- What does [insert response option] mean for you?
  - How often would you say you have bowel movements? Daily? X times a week? If you were to answer this this time and day last week, would your answer have changed?

Screen 5


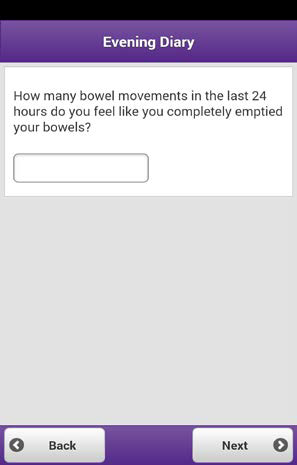


- What does this question mean to you? Can you put the question into your own words?
  - How is this question different than the last question you just answered?
  - What does feeling “like you completely emptied your bowels” mean to you? Are there times when you feel like you have not completely emptied? What does that feel like?
- Is the meaning of the question clear or unclear to you? If unclear, why is it unclear?
- I see you selected [patient’s response]. In the last question about number of bowel movements in the last 24 hours, you said [patient’s response/number]. What made you choose the number for this question?
- What does [insert response option] mean for you?
  - How often would you say you have bowel movements where you don’t feel completely emptied? Does it change from day to day? Week to week? What makes it change (e.g., foods, stress)?

Screens 6 and 7


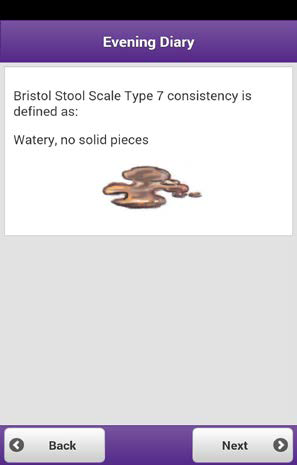

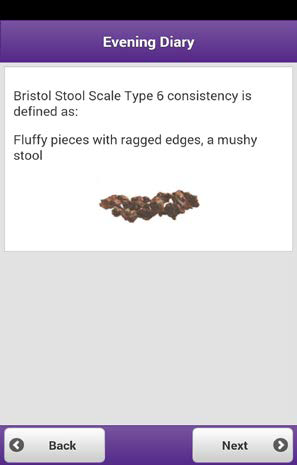


• What are the two screens showing you?

• Is the description of the type of stool clear or unclear? If unclear, how would you change it

to be more clear?

Screen 8


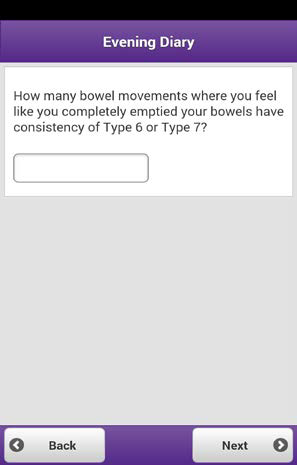


- What is this question asking you? Can you put the question into your own words?
  - What does “consistency of Type 6 or Type 7” mean to you?
  - Can you remember what Type 6 was? And Type 7?
  - What does it mean when it says “bowel movements where you feel like you completely emptied your bowels”? Does your answer change reading it again this time?
- Is the meaning of the question clear or unclear to you? If unclear, why is it unclear?
- What does [insert response option] mean for you? What made you select that number?
  - Do you ever experience bowel movements that you feel like you’ve completely emptied, but were Types 6 or 7? If yes, how often?

Screens 9–12

- [For each screen, Screens 9-12] What is this question asking you? Can you put the question into your own words?
  - I understand you may not be taking any drugs for your FD at the moment. Let’s say you were in new clinical study where you were instructed to take a medication each morning and evening. Would you be able to answer these questions accurately every day for few months?
- Is the meaning of the question clear or unclear to you? If unclear, why is it unclear? Would you change anything about the wording?

Screens 13–16


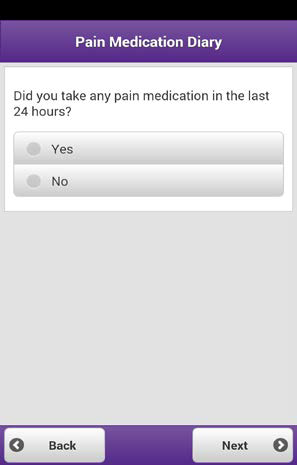

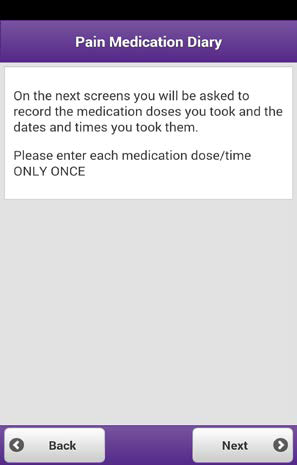


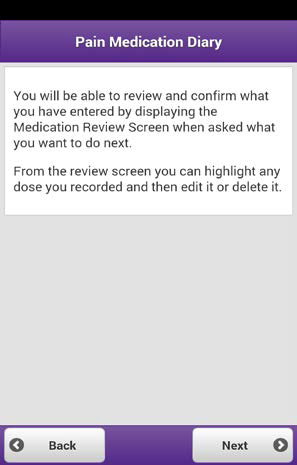

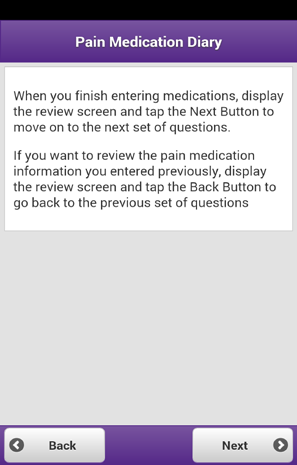


- [For screen 13] What is this question asking you? Can you put the question into your own words?
  - How would you define a “pain medication”?
  - If you were in a clinical trial, you may be asked this question if you take anything for pain in addition to the study medication. Is the meaning of the question clear or unclear to you? If unclear, why is it unclear? Would you change anything about the wording?
- [For each screen, Screens 13-16] What is this screen instructing you to do?

Screens 17–24


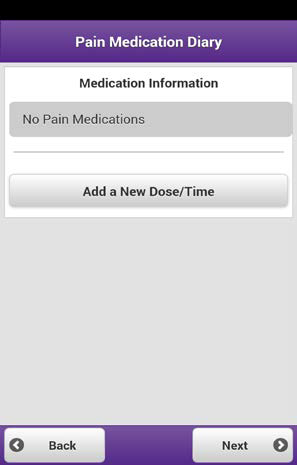

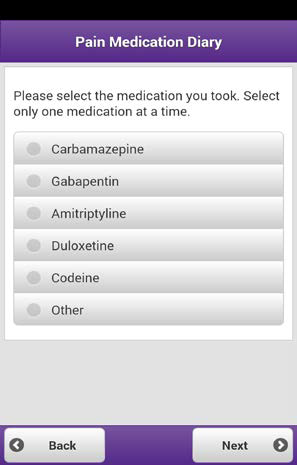


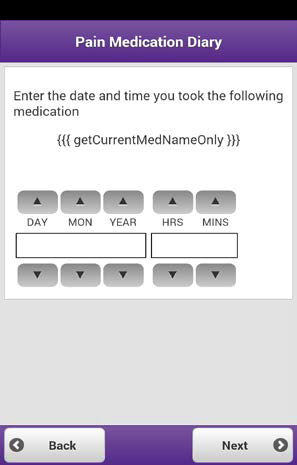

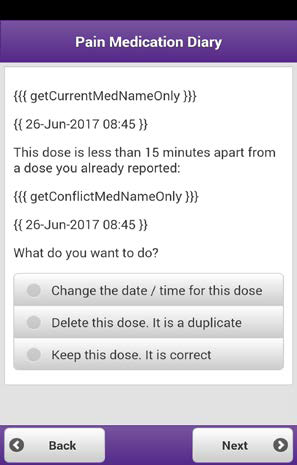

- [For screen 18] What is this screen instructing you to do?
  - Are you familiar with any of these medications on this list?
    - [If yes] Which ones? How often do you use it?
    - [If no] These are medications that can be used for pain when the pain is particularly bad. If you were in clinical trial and you had to take any of these pain medications in addition to investigative study medication, would you be able to report it accurately?
- [For each screen, Screens 17-24] What is this screen showing you or instructing you to do? Is anything unclear? Would you change anything about this screen?
- [For screen 20] In this screen, where it says “{{{ getCurrentMedNameOnly }}}”, it refers to the specific pain medication that you have taken and reported. What do each of the responses (“Change the date”; “Delete”; and “Return”) mean to you?

Screens 25 and 26


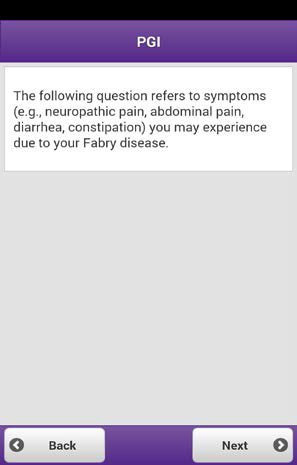


- [For screen 25] What is this screen showing you?
  - Are there other symptoms that you would include as an example, and if so what are they?
- [For screen 26] What is this question asking you? Can you put it in your own words?
  - What does “overall severity of the symptoms” mean to you?
  - What does “over the past 7 days” mean to you?
  - What made you select [patient’s response]? Which specific symptoms are you thinking about? Are you thinking about any one specific symptom more than the other?
- Can you think back to a time when you would’ve selected a different answer [go up and down the scale, relative to patient’s response]? How were you feeling at the time?
  - - What does this response option mean to you?

Screens 27 and 28


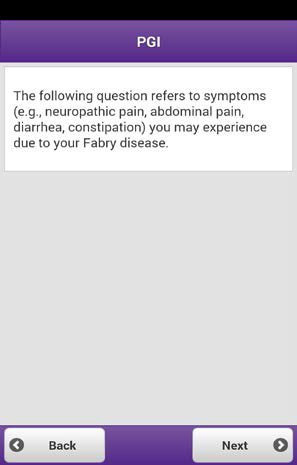

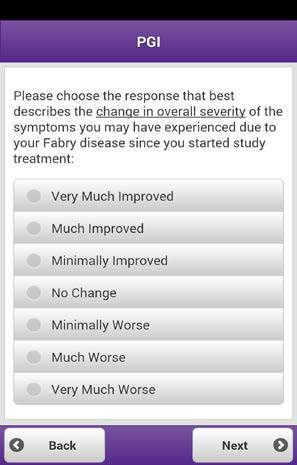


- [For screen 27] What is this screen showing you?
- [For screen 28] What is this question asking you? Can you put it in your own words?
  - What does “change in overall severity” mean to you?
    - What does this response option mean to you?
  - I understand this question may not apply to you since you were not in a study that involved a treatment. Let’s imagine that you were in a study and you took a medication for a few months. Would you be able to answer this question accurately based on how you felt?
  - If the treatment was working, which answer is the ideal answer that you would have chosen? If there was “no change”, would you take the drug again? If there was “minimally improved” change, would you take the drug again?

Screens 29 and 30


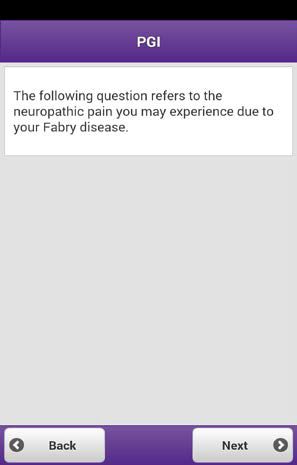

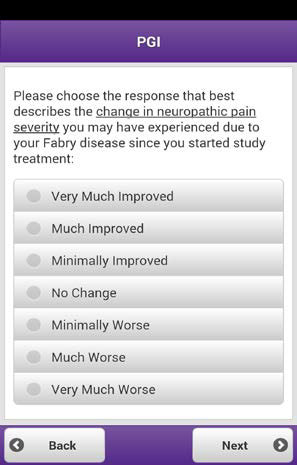


- [For screen 29] What is this screen showing you?
  - How is it different than the one you saw few screens back?
- [For screen 30] What is this question asking you? Can you put it in your own words?
  - Again, how is it different than the question you just answered a couple of screens back?
  - What does “change in neuropathic pain severity” mean to you?
    - What does this response option mean to you?
  - Again, let’s make believe that you were in a study and you took a medication for a few months. Would you be able to answer this question accurately based on how you felt?
  - If the treatment was working, which answer is the ideal answer that you would have chosen? If there was “no change”, would you take the drug again? If there was “minimally improved” change, would you take the drug again?

**S2.2 Questions posed to patients during the device usability testing interview**

- How easy or difficult was it to use the device to answer the questions? Please explain.
- On a scale of 1 to 5, where 1 = poor and 5 = excellent, how would you rate…
  - The sensitivity of using your finger to operate the device?
    - What made you choose that answer?
  - The appearance of the instructions, questions and response options on the device screens?
    - What made you choose that answer?
- On a scale of 1 to 5, where 1 = difficult and 5 = easy, how would you rate…
  - Your ability to select an answer on the device?
    - What made you choose that answer?
  - Your ability to advance to the next screen on the device?
    - What made you choose that answer?
  - The readability of the font size on the device screens?
    - What made you choose that answer?
  - The overall ease of use of the device?
    - What made you choose that answer?
- Would you be able to answer these questions accurately every day for few months? How would you find doing it everyday?
- Do you have any additional feedback to share on the usability of the device?

**Supplementary Figure**


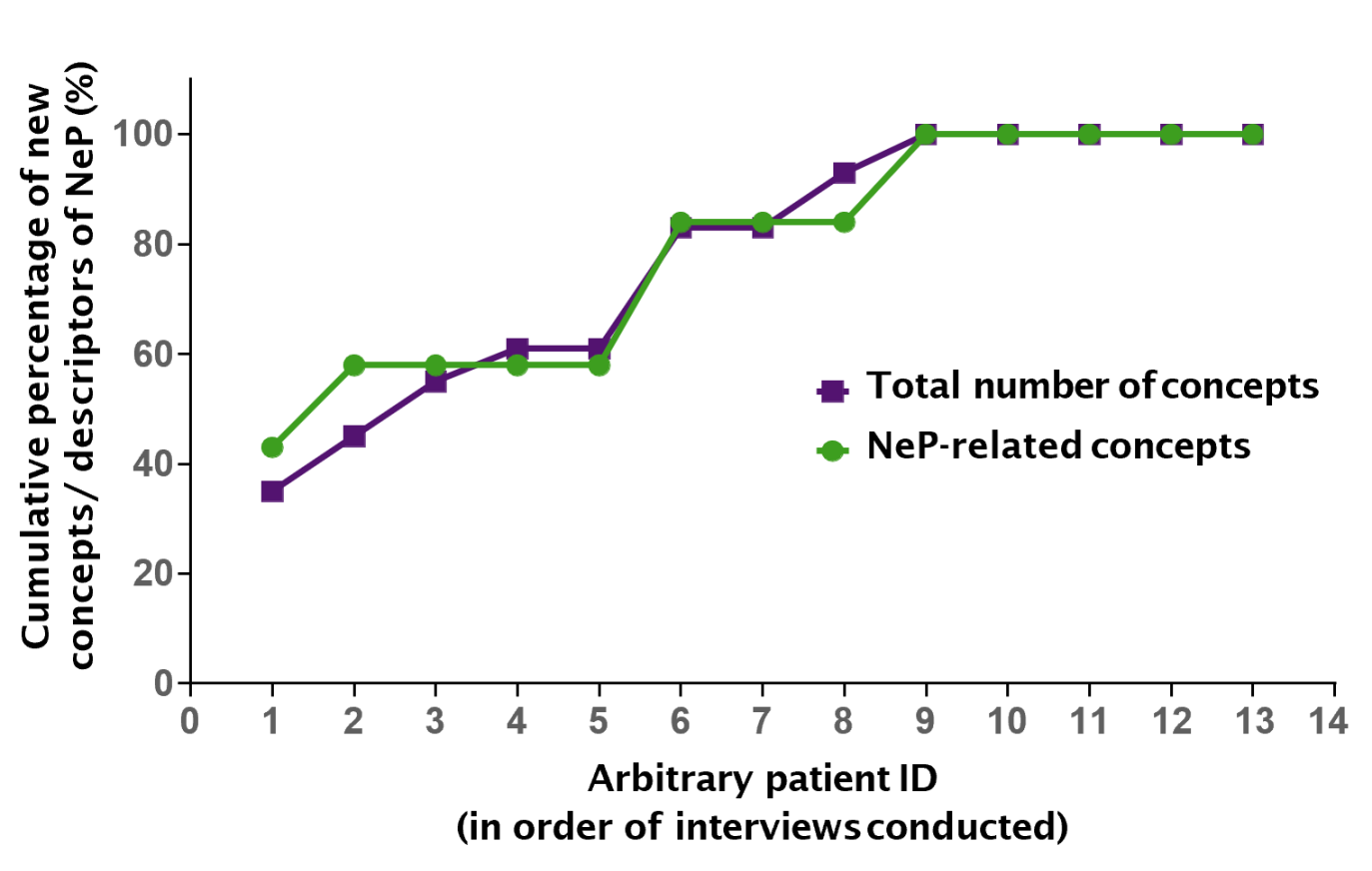


**Fig. 1S:** Concept occurrence of all unique concepts and neuropathic pain-related concepts (sensations/descriptors of neuropathic pain). NeP, neuropathic pain.
